# Exploring the Potential of Phytocannabinoids Against Multidrug-Resistant Bacteria

**DOI:** 10.3390/plants14131901

**Published:** 2025-06-20

**Authors:** Carmina Sirignano, Simona De Vita, Ernesto Gargiulo, Massimiliano Lucidi, Daniela Visaggio, Maria Giovanna Chini, Gianluigi Lauro, Giuseppina Chianese, Paolo Visca, Giuseppe Bifulco, Orazio Taglialatela-Scafati

**Affiliations:** 1 Department of Pharmacy, University of Naples Federico II, Via Domenico Montesano 49, 80131 Napoli, Italy; carmina.sirignano@unina.it (C.S.); ernesto.gargiulo@unina.it (E.G.); scatagli@unina.it (O.T.-S.); 2 Department of Pharmacy, University of Salerno, Via Giovanni Paolo II 132, 84084 Fisciano, Italy; sdevita@unisa.it (S.D.V.); glauro@unisa.it (G.L.); 3 Department of Science, Roma Tre University, Viale Guglielmo Marconi 446, 00146 Rome, Italy; massimiliano.lucidi@uniroma3.it (M.L.); daniela.visaggio@uniroma3.it (D.V.); paolo.visca@uniroma3.it (P.V.); 4 Department of Biosciences and Territory, University of Molise, Contrada Fonte Lappone, 86090 Pesche, Italy; mariagiovanna.chini@unimol.it

**Keywords:** multidrug resistance, cannabinoids, inverse virtual screening, antibacterial activity

## Abstract

The rapid emergence of multidrug-resistant (MDR) bacterial pathogens poses a critical threat to global health, creating an urgent need for novel antimicrobial agents. In this study, we evaluated a small library of natural and semisynthetic phytocannabinoids against a broad panel of MDR Gram-positive bacterial strains, evidencing very good activity in the low µM range. We provide evidence of the antibacterial activity of the two separated enantiomers of cannabidiol, offering novel insights into the stereochemical aspects of their bioactivity. To investigate the possible molecular targets and clarify the mechanism of action, we employed Inverse Virtual Screening (IVS), a computational approach optimized for predicting potential protein–ligand interactions, on three selected MDR bacterial species. Interestingly, key targets belonging to important bacterial metabolic pathways and defense mechanisms were retrieved, and the results were used to rationalize the observed biological activities. To the best of our knowledge, this study marks the first application of IVS to microorganisms, offering a novel strategy for identifying bacterial protein targets. The results pave the way for future experimental validation, structure-based drug design, and the development of novel antibacterial agents.

## 1. Introduction

The rise of multidrug resistance (MDR) among infectious pathogens represents one of the most critical challenges to global public health. Defined as the ability of microorganisms to withstand the effects of multiple antimicrobial agents, MDR complicates treatment protocols and contributes to increased morbidity, mortality, and healthcare costs worldwide [1]. Bacteria, fungi, and even some viruses have developed resistance mechanisms, weakening the efficacy of drugs traditionally used for infectious disease management. Among bacterial pathogens, notable MDR examples include methicillin-resistant *Staphylococcus aureus* (MRSA), carbapenem-resistant *Enterobacteriaceae* (CRE), and vancomycin-resistant *Enterococci* (VRE), which collectively account for a significant portion of nosocomial and community-acquired infections [2]. The evolution and spread of MDR pathogens are driven by a range of factors, including the overuse and misuse of antimicrobials in healthcare and inadequate infection control practices within healthcare settings. These factors not only promote the selection of resistant strains but also facilitate their transmission within and between communities.

The COVID-19 pandemic has highlighted the escalating global threat of antimicrobial resistance (AMR), with the increased use of antibiotics—often inappropriately, even in the presence of low rates of bacterial co-infection—intensifying the prevalence of MDR organisms. This increase in AMR not only exacerbated patient morbidity and mortality but also placed a substantial economic burden on healthcare systems worldwide, underscoring the urgent need for sustainable antimicrobial stewardship and global policy reforms to mitigate the spread of resistant infections [3].

Furthermore, the limited development of new antimicrobial agents magnifies the issue, with fewer therapeutic options available to fight resistant infections, particularly in resource-limited settings [4]. Addressing AMR requires a comprehensive approach that integrates careful management of existing antimicrobials, improved diagnostic techniques, and the development of novel antimicrobial agents. Surveillance and public health initiatives are equally critical to monitor and mitigate the spread of MDR pathogens globally [5]. Natural products, particularly plant secondary metabolites, can play an important role in combating MDR pathogens due to their diverse structures and potential to inhibit resistance mechanisms like efflux pumps, and to synergize with existing antibiotics. Their multifaceted modes of action make them promising candidates for addressing the global antibiotic resistance crisis [6].

The Antibacterial properties of phytocannabinoids have been recognized since the 1950s, with early reports highlighting the antiseptic and antitubercular effects of *Cannabis sativa* L. preparations for oral and topical applications. Subsequent studies, including the work by Appendino et al. in 2008 [7], evidenced the antibacterial potential of compounds such as cannabidiolic acid (CBDA), cannabidiol (CBD), cannabichromene (CBC), cannabigerol (CBG), and Δ^9^-tetrahydrocannabinol (THC), revealing their activity against resistant bacterial strains and conducting preliminary structure–activity relationship studies [7]. Currently, two Phase II clinical trials are investigating CBD-based formulations for their effectiveness in treating bacterial skin infections. These studies aim to leverage CBD antimicrobial, anti-inflammatory, and immune-modulating properties, which have been demonstrated in preclinical models [8]. In addition, a recent work suggested that the formulation of topical products with active concentrations of CBG and/or CBD may offer a viable solution for managing skin conditions where microorganisms and inflammation are significant etiological agents, including psoriasis, atopic dermatitis, and acne [9]. Recent analyses investigated the antibacterial activity of CBG [10] and other phytocannabinoids [11,12,13]. Although these studies hypothesize that the mechanism by which phytocannabinoids fight bacterial infections is associated with a disruption of the bacterial membrane, further investigations are required to unambiguously elucidate phytocannabinoid molecular targets responsible for their antimicrobial activity.

**Figure 1:**
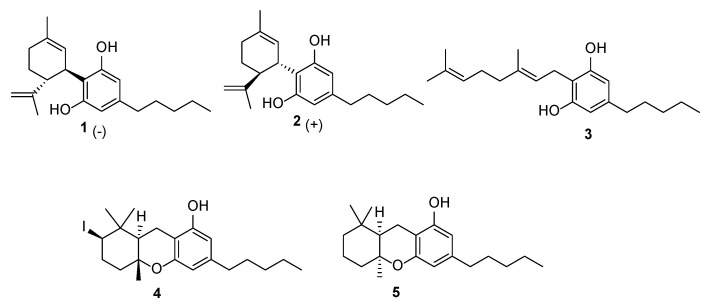
Chemical structures of the phytocannabinoids investigated.

The present study aimed to evaluate a small library of natural and semisynthetic phytocannabinoids (Figure 1), available in our laboratories, against a broad panel of MDR bacterial strains, including several previously untested strains, which were primarily focused on *S. aureus*. To understand the mechanisms of action, we employed the robust and efficient in silico Inverse Virtual Screening (IVS) technique, which we have developed and optimized, representing a key tool for predicting the molecular targets of test compounds [14,15,16,17]. IVS is used to predict potential protein/ligand complexes by simulating the interaction of the compounds with a broad range of proteins, allowing us to gain deeper insights into their biological activity, hypothesize the mechanism of action, and discover novel therapeutic pathways [18]. To date, only a few studies have reported “direct” molecular docking (i.e., many ligands vs. one or few targets) experiments involving, at most, a limited number of bacterial targets. Notably, this study represents the first application of IVS to microorganisms, marking a significant breakthrough in computational target identification and enabling a broad-scale evaluation of antibacterial activity.

## 2. Results and Discussion

### 2.1. Screening of Phytocannabinoids Against Multidrug-Resistant Pathogens

In the present study, we investigated the antibacterial activity of a small library of natural and semisynthetic phytocannabinoids (Figure 1) available in our laboratories from previous studies [19] to determine their potential against MDR bacterial strains. In particular, to evaluate the impact of stereochemistry on bioactivity, we included in the library the two enantiomers of CBD (**1** and **2**). (−)-CBD [20] is a major constituent of fiber hemp with a biological profile of therapeutic importance, including its effectiveness in treating genetic forms of juvenile epilepsy [21]. Interestingly, (+)-CBD, prepared in our lab following the Hanus protocol [22], exhibited a different binding profile to CB_1_ and CB_2_ receptors, supporting the notion that the chirality of phytocannabinoids significantly influences their pharmacological effects. Moreover, since preliminary structure–activity relationships (SARs) revealed that the terpene moiety significantly influences antibacterial potency [23], we included in the library CBG (**3**) and two of its cyclized semisynthetic derivatives, **4** and **5**, described in our previous work [19].

An initial screening to test the antibacterial activity of phytocannabinoids was conducted on *Escherichia coli* MG1655 and *S. aureus* ATCC 25923, selected as prototypic Gram-negative and Gram-positive bacteria, respectively. This preliminary assessment revealed that all tested compounds exhibited minimum inhibitory concentration (MIC) values ≤ 8 μg/mL against *S. aureus*, indicating significant antibacterial activity, but no notable effects were observed on *E. coli* growth (MIC > 64 μg/mL). As inhibitory effects were observed exclusively against *S. aureus*, we expanded our analysis to a broader panel of Gram-positive bacteria to better define the antibacterial spectrum of the tested compounds (Table 1).

These strains include *Enterococcus faecalis* and *E. faecium*, which cause a wide variety of infections, including endocarditis, urinary tract infections, prostatitis, intra-abdominal infections, and wound infections. All compounds proved to be markedly active against all tested Gram-positive bacteria, showing MIC ≤ 8 μg/mL, except for compound **4**, which showed a MIC > 64 μg/mL only for *S. aureus* ATCC 43300 (Table 2).

The minimum bactericidal concentration (MBC) was determined for *S. aureus* ATCC 25923. All compounds, except compound **4**, demonstrated bactericidal activity at concentrations two- to four-fold higher than their MIC values, highlighting their potential as effective antibacterial agents. In contrast, compound **4** showed no bactericidal effect even at the highest concentration tested (MBC > 64 μg/mL).

The stereochemistry of CBD enantiomers (**1** and **2**) influences their antibacterial activity, suggesting the involvement of a specific molecular target. Except against *E. faecium* BM4147, the natural enantiomer (−)-*trans* CBD (**1**) was generally more active than **2**. CBG (**3**) exhibited notable efficacy on all the tested Gram-positive strains and, to the best of our knowledge, this is the first report on the antibacterial effects of CBG against *Enterococcus* spp. Semisynthetic derivatives **4** and **5** negatively impacted this activity, highlighting the importance of modifications of the terpene moiety [23].

To evaluate the cytolytic activity of the tested phytocannabinoids, we assessed hemolysis in erythrocytes from healthy donors across three blood groups (A+, B+, and 0-). All compounds demonstrated negligible hemolytic activity (<3% hemolysis, Table S1). The in vivo toxicity was further assessed using *Galleria mellonella* larvae as a model organism. Compared to cell lines, insect larvae provide a more physiologically relevant model for the assessment of toxicity due to their complex detoxification systems [27]. Only CBG (**3**) significantly reduced larval survival to below 80%, indicating in vivo toxicity (Figure S1), in contrast with previous reports [10]. This discrepancy may stem from differences in experimental conditions or the selected animal model.

In summary, our findings demonstrated the promising antibacterial activity of selected phytocannabinoids, particularly against MDR Gram-positive pathogens. To analyze molecular targets and SAR, we employed, for the first time, Inverse Virtual Screening (IVS) methodologies.

### 2.2. Inverse Virtual Screening (IVS)

The IVS methodology was used to select the most probable binding partners for compounds **1**–**5**. Briefly, this methodology relies on molecular docking experiments in which a small set of compounds is screened against a panel of proteins that share structural or functional features or belong to the same organism, as in this case (*vide infra*). The prediction of the ligand–protein interactions is performed through a normalization phase that involves the same process using a set of decoy molecules (i.e., compounds resembling the physicochemical properties of the studied compounds but with different chemical structures) [14,15,16,17]. Specifically, the binding affinity of a single compound for a specific target is divided by the average of the binding affinities of the decoys for the same target, providing a quantitative new parameter which we named “V” (see Section 3for further details). In this way, the predicted molecular complexes are ranked by the adimensional V value, providing valuable insights to explain the biological mechanisms of action of these compounds.

To gain a deeper understanding of the antimicrobial activity observed, we selected three representative bacterial species from those tested: *S. aureus*, *B. spizizenii*, and *E. faecium*. One of the key challenges in this approach was the limited availability of experimental structures in the Protein Data Bank (PDB) and the absence of predicted models in AlphaFold. To circumvent this limitation, we broadened our scope to include all available protein structures from the main species, regardless of strain, as done in other papers [28,29]. This allowed us to construct, through an automatic workflow developed by us [30], comprehensive protein panels consisting of 3790, 41, and 135 elements for *S. aureus*, *B. spizizenii*, and *E. faecium*, respectively. As previously mentioned, this study represents the first application of such a complex and integrative computational approach to bacterial systems. After the normalization phase, the most promising targets were preliminarily selected by considering a V value above 1.0 and a ligand binding affinity below −6.0 kcal/mol (see Section 3).

#### 2.2.1. Rationalization of the Results on *S. aureus*

With these premises, we started our rationalization from metabolites **1**–**3** to establish a benchmark for the evaluation of semisynthetic compounds like **4** and **5**.

**Figure 2:**
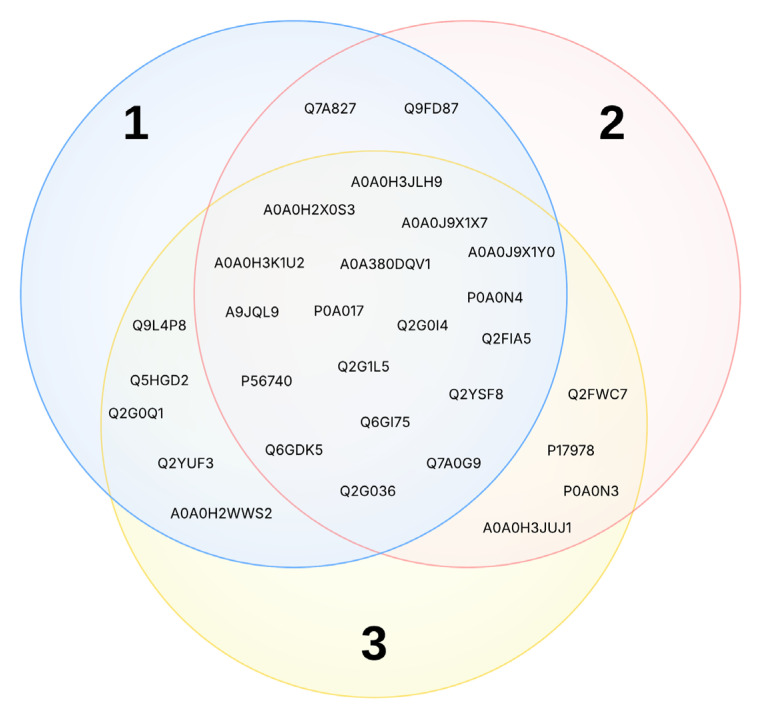
The UniProt ID of the target that emerged from the normalization of the results of IVS on compounds **1**–**3**.

The analysis of the IVS data revealed that several targets are shared among the three metabolites (Figure 2 and Table S2). Notably, the targets can be grouped into two main macro-categories: proteins involved in metabolism and the biosynthesis of fundamental macromolecules, and proteins associated with detoxification and defense mechanisms. To gain a more detailed view, the targets were further grouped according to their biological pathways:

a. Fatty acid metabolism

Specifically, the enoyl-[acyl-carrier-protein] reductase FabI (Q6GI75, A0A0H3JLH9, A0A0J9X1X7, A0A0J9X1Y0) is a key enzyme in the fatty acid synthesis pathway and catalyzes the final reduction step in the elongation cycle of fatty acid biosynthesis [31]. Inhibition of this enzyme can lead to the interruption of membrane synthesis. Additionally, the enantioselective difference between compounds **1** and **2** may arise from specific interactions within the FabI active site [32], which catalyzes a stereospecific reduction of the double bond in a fatty acid chain, influencing their binding affinity. Furthermore, the longer terpene chain of compound **3** could enhance hydrophobic interactions with FabI, potentially contributing to the high observed activity. The HMG-CoA synthase (A0A0H3K1U2, Q9FD87) is a key enzyme in the mevalonate pathway, utilizing a Claisen condensation between acetyl-CoA and acetoacetyl-CoA, thereby playing a crucial role in the production of prenylated lipids necessary for maintaining cell membrane integrity [33]. Inhibition of this enzyme disrupts membrane homeostasis, ultimately leading to bacterial cell death.

b. Nucleotide biosynthesis

The adenylosuccinate lyase (Q7A0G9) catalyzes two key enzymatic reactions involved in the de novo purine biosynthesis pathway, which serve as building blocks for DNA and RNA synthesis. Given its important function in nucleotide metabolism, inhibition of this enzyme directly impacts DNA replication, ultimately preventing correct cell division. This disruption in the purine biosynthetic pathway may explain the observed bacteriostatic effect [34]. Bifunctional metallophosphatase/5′-nucleotidase (Q2G1L5) is involved in nucleotide recycling and phosphate metabolism. The inhibition of this enzyme can lead to nucleotide depletion, undermining DNA replication and RNA synthesis [35]. Given these broad-spectrum effects on nucleotide metabolism, this target could be significant for inhibiting bacterial growth, especially in rapidly dividing cells.

c. Folate metabolism

Dihydrofolate reductase (P0A017) and the dihydroneopterin aldolase (P56740) are both involved in folate metabolism [36,37]. Targeting this pathway is a well-established strategy in antibacterial therapy. For instance, trimethoprim acts as a competitive inhibitor of bacterial dihydrofolate reductase, thereby blocking the conversion of dihydrofolate to tetrahydrofolate, a key step in the synthesis of nucleotides and amino acids. This selective inhibition is possible thanks to the significant structural differences between the prokaryotic and human forms of DHFR, allowing the design of compounds that preferentially bind the bacterial enzyme [37].

d. Vitamin and cofactor biosynthesis

Pantothenate synthetase (Q6GDK5) catalyzes the biosynthesis of pantothenic acid (vitamin B5), a precursor for coenzyme A (CoA), which is, in turn, essential for lipid metabolism. Inhibiting pantothenate synthesis disrupts bacterial fatty acid biosynthesis and energy metabolism [38]. Ketol-acid reductoisomerase (Q2YUF3) plays a role in the biosynthesis of branched-chain amino acids (BCAAs), including valine, leucine, and isoleucine. Given that BCAAs are essential for bacterial survival but are absent in humans, this enzyme could represent an attractive antibacterial target [39]. Since vitamins and cofactors are essential for bacterial metabolism, targeting their biosynthetic pathways could represent a valid strategy for antibacterial agents.

e. Redox homeostasis

Betaine-aldehyde dehydrogenase (A0A0H2X0S3), coenzyme A disulfide reductase (Q2FIA5), and 4,4′-diapophytoene synthase (A9JQL9) play critical roles in oxidative stress response and redox balance, protecting bacterial cells from reactive oxygen species. In detail, the first one is a NAD(P)^+^-dependent enzyme that produces glycine betaine as an osmoprotectant [40], whereas coenzyme A disulfide reductase plays a relevant role in maintaining intracellular redox homeostasis [41]. The 4,4′-diapophytoene synthase (CrtM) is involved in the biosynthesis of carotenoid pigments, the staphyloxanthin, which protects bacteria from oxidative stress. In detail, it condensates two molecules of farnesyl diphosphate (FPP) to obtain the 4,4′-diapophytoene (dehydrosqualene) [42].

FAD-containing oxidoreductase (Q2G0I4) is involved in electron transfer reactions, which are decisive for the bacterial respiratory cycle and metabolism [43]. Finally, cytochrome P450 (A0A380DQV1) is involved in oxidative metabolism, drug detoxification, and lipid modification. Particularly, it plays a key role in sterol and lipid metabolism, contributing to membrane stability, and therefore, its inhibition can lead to disruptions in sterol biosynthesis [44]. Disrupting these enzymes may increase ROS accumulation, making bacteria more susceptible to host immune defenses.

f. Transcriptional regulation

HTH-type transcriptional regulator QacR (P0A0N4) is a regulatory protein involved in antibiotic resistance mechanisms; particularly, it binds to the promoter of the gene that is responsible for the expression of the multidrug efflux pump QacA. Interrupting its action could lead to an uncontrolled expression of efflux pumps, which would deplete the bacterium’s energetic pool [45].

The broad spectrum of pathways affected, including fatty acid biosynthesis, nucleotide metabolism, transcriptional regulation, folate synthesis, redox balance, membrane integrity, and amino acid biosynthesis, could rationalize the good activity against *S. aureus*.

Moving on to semisynthetic molecules **4** and **5**, the first one showed unpaired biological data in *S. aureus*. Indeed, it proves ineffective against the ATCC 43300 strain while exhibiting a certain degree of inhibition in the ATCC 25923 strain, making it challenging to rationalize its mechanism of action unequivocally (Table S3). However, at this stage, no clear hypothesis can be made concerning the reasons underlying the different activities encountered in the two bacterial strains since no 3D structure specifically assigned to each strain (i.e., ATCC 43300 and ATCC 25923) is available in the Protein Data Bank. Compound **5**, despite its more rigid and bulky structure compared to compounds **1**–**3**, exhibits promising binding affinity toward some of the same proteins identified for those compounds, suggesting that its activity can be attributed to interactions with these targets (Table S4).

#### 2.2.2. Rationalization of the Results on *E. faecium*

The complete results emerging from the IVS and the selection of the most promising targets for compounds **1**–**5** are reported in the Supporting Material (Tables S5–S10). Among the active compounds, except for **4**, the docking studies identified a set of shared targets that are consistently highlighted and are likely to be involved in bacterial survival. For instance, targets such as I3U4H4, Q3XX76, and Q3Y316 were frequently retrieved in the interaction profiles of the active compounds.

To further refine these results, a more detailed assessment of molecular docking poses was carried out. This secondary analysis aimed at evaluating the binding interactions at a structural level, prioritizing targets that demonstrated strong and well-defined ligand–protein interactions. As a result of this qualitative and quantitative evaluation, the initial broad selection of potential targets was narrowed down, leading to a final selection of the most relevant protein partners. The refined list of best-interacting targets for each compound is reported in Table 3.

A comparative analysis of the selected compounds highlighted key trends in target engagement. In detail, the natural CBD and CBG (**1**–**3**) interacted with I3U4H4, Q3XX76, and P50870, which appear to be critical molecular targets contributing to antimicrobial activity. Specifically, I3U4H4 is a serine hydroxymethyltransferase that uses tetrahydrofolate (THF) as the one-carbon carrier to catalyze the reversible interconversion of serine and glycine and is essential for the production of purines, thymidylate, methionine, and other significant macromolecules [46]. Its inhibition, therefore, could disrupt critical metabolic pathways, impairing bacterial proliferation. Q3XX76 cleaves peptides in various proteins in a process that requires ATP hydrolysis. It plays a significant role in the degradation of misfolded proteins and facilitates the recycling of amino acids and the removal of toxic aggregates that accumulate under stress conditions [47,48]. Finally, P50870 contributes to antibiotic resistance by acetylating streptogramin A compounds, thereby inactivating these antibiotics [49,50].

Concerning the semisynthetic derivatives **4** and **5**, both compounds showed significant inhibitory activity with MIC values of 1 µg/mL, but the number of the putative targets selected through IVS was lower compared to natural analogs, especially in the case of compound **4**. The iodine substituent in compound **4** may have influenced steric and electronic properties, possibly affecting binding affinity and the IVS outcomes. Compound **5**, on the other hand, retained two of the key targets pointed out for **1**–**3** (i.e., I3U4H4 and P50870), suggesting that its antimicrobial mechanism may still rely on disrupting essential metabolic pathways and resistance mechanisms.

#### 2.2.3. Rationalization of the Results on *B. spizizenii*

As already stated, the limited availability of crystallographic structures for this bacterial species posed a significant challenge to the identification of putative protein partners for these compounds. The Protein Data Bank (PDB), indeed, contains only a few experimental structures for this organism, which prevented a broad molecular docking campaign.

Following the same principles for *S. aureus* and *E. faecium*, we first considered compounds **1**–**3**. The targets emerging from each compound were compared, and among the predicted targets, only one was shared by all three metabolites: the iron-containing alcohol dehydrogenase YugJ (UniProt ID: O05239). This enzyme plays a crucial role in microbial metabolism, particularly in the oxidation–reduction of alcohols and aldehydes, which are essential for cellular homeostasis and survival. After comparing these results with those obtained for the semisynthetic compounds (**4** and **5**), it emerged that the most promising binding partner for these compounds was the same alcohol dehydrogenase.

From the visual inspection of the binding poses, all the molecules considered respect the interaction with amino acids deemed fundamental for the binding with NADP(H) [51].

**Figure 3:**
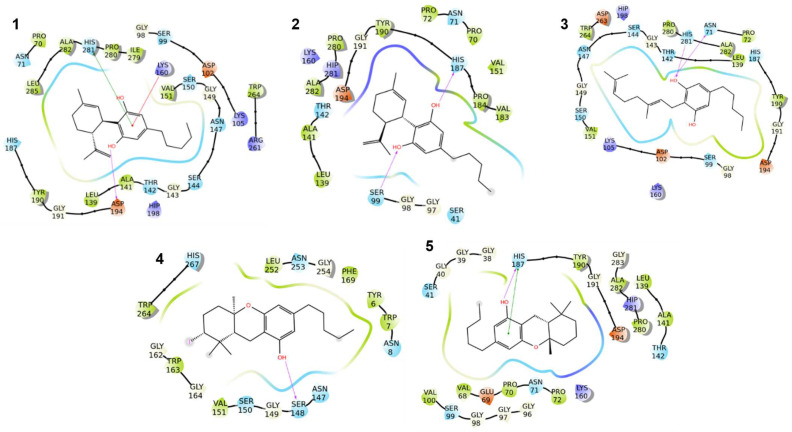
Two-dimensional interaction diagram of compounds **1**–**5** with alcohol dehydrogenase YugJ. Pink arrows represent the hydrogen bonds, and the π-π stackings are represented by green lines. Polar residues are in cyan, hydrophobic ones are in green, and red or blue represent negatively and positively charged amino acids, respectively.

A closer examination of the molecular docking poses (Figure 3) revealed that the compounds established direct and proficient contacts, such as hydrogen bonds or π-π stackings, with key amino acids within the enzyme binding cavity (e.g., Ser99, His187, and His281), which are positioned in the NADP(H)-binding region. These interactions suggest a probable competitive inhibition mechanism, where the compounds may effectively displace the natural cofactor and interfere with the catalytic activity of the enzyme. Additionally, some compounds formed hydrophobic interactions with residues crucial for NADP(H) stabilization, further reinforcing their ability to act as competitive inhibitors. This combination of hydrogen bonding, π-π stacking, and hydrophobic interactions enhances the binding affinity and suggests a robust inhibition profile. Given the importance of alcohol dehydrogenase in cellular metabolism, its inhibition could impact bacterial physiological processes, potentially impairing growth and survival. These findings underscore the therapeutic potential of these molecules and urge further investigation to assess their efficacy in biological systems.

#### 2.2.4. Implications for Bacterial Multidrug Resistance

Bacterial multidrug resistance is often associated with a combination of various biochemical and genetic variations that enable bacteria to evade the effects of antimicrobial agents [52]. Several of the proteins selected through the IVS procedures could be linked to the effects of these molecules on resistant bacterial strains.

In *S. aureus*, the enoyl-[acyl-carrier-protein] reductase FabI has emerged as a promising alternative target to overcome resistance mechanisms, particularly in methicillin-resistant *S. aureus* (MRSA). Unlike FabK, FabI is highly conserved in *S. aureus*, making it an attractive target for novel antibacterial agents [53,54]. Two inhibitors, afabicin and nilofabicin, have recently completed phase IIa clinical trials, demonstrating the global interest in this inhibitory strategy [54]. Beyond FabI inhibition, alternative strategies to target MRSA have been explored, including the use of statins, the most well-known HMG-CoA synthase inhibitors, to prevent the synthesis of the isoprenoids required in the bacterial cell wall and make the cell sensitive to reactive oxygen species. Interestingly, the modulation of HMG-CoA synthase, singled out by our IVS studies, affects the membrane composition and the functional membrane microdomains (FMMs) in prokaryotes, which are similar to membrane lipid rafts, where bacterial processes are compartmentalized. In MRSA, key resistance-related proteins, including PBP2a (the alternative penicillin-binding protein responsible for methicillin resistance), PBP2 (which participates in cell wall synthesis), and PrsA (a chaperone involved in protein folding), are part of the FMM cargo [54]. The integrity and proper organization of these microdomains are critical for the oligomerization and activity of PBP2a, suggesting that disrupting membrane composition could impair the resistance mechanism.

Another intriguing target, retrieved through IVS studies, is the transcriptional regulator QacR, which controls the expression of multidrug efflux pumps. Typically, antibiotic resistance strategies aim to inhibit efflux pumps to increase intracellular drug retention. However, an alternative approach could involve disrupting QacR activity, resulting in the uncontrolled overexpression of efflux pumps. This excessive efflux activity could deplete bacterial energy stores, ultimately compromising bacterial survival [45].

In *E. faecium*, resistance to vancomycin poses a significant clinical challenge. Alternative targets such as serine hydroxymethyltransferase (SHMT) and streptogramin A acetyltransferase, emerging from our in silico approach, have been identified as promising intervention points to combat vancomycin-resistant *E. faecium* (VRE) infections [46,55,56]. SHMT plays a crucial role in one-carbon metabolism, which is essential for nucleotide synthesis and bacterial survival. Inhibiting this enzyme could impair bacterial replication and increase susceptibility to antibiotics. Meanwhile, streptogramin A acetyltransferase confers resistance to streptogramin-class antibiotics, a last-resort option for VRE treatment.

Ultimately, considering the limited availability of *B. spizizenii* crystal structures and our protocol based on the qualitative and quantitative analysis, our outcomes pose a significant challenge to a comprehensive evaluation. However, we hypothesize that the iron-containing alcohol dehydrogenase YugJ could represent a valuable target for rationalizing the antimicrobial observed activity. All these findings provide interesting preliminary information for further investigations into the mechanism of action of the natural or semisynthetic cannabinoids against Gram-positive bacteria.

## 3. Materials and Methods

### 3.1. Phytocannabinoids

(−)-CBD and CBG were obtained from our previous studies [57]. (+)-CBD was prepared in our lab following the Hanus protocol [22]. Compounds **4** and **5** are two semisynthetic products obtained by reacting CBG with iodine, as previously reported [19].

Compound **4**. Pale yellow amorphous solid. [α]_D_ = 0. ^1^H NMR (CDCl_3_, 500 MHz) δ 6.23 (1H, s, H-4), 6.17 (1H, s, H-2), 4.24 (1H, dd, *J* = 12.8, 3.8 Hz, H-6′), 2.83 (1H, dd, *J* = 16.5, 4.9 Hz, H-1′a), 2.45 (1H, overlapped, H-5′a), 2.44 (1H, overlapped, H-1′b), 2.44 (2H, overlapped, H-1″), 2.33 (1H, dd, *J* = 13.6, 3.8 Hz, H-5′b), 1.86 (1H, dd, *J* = 11.5, 3.6 Hz, H-2′), 1.82 (1H, m, H-4′a), 1.75 (1H, bdd, *J* = 13.6, 3.8 Hz, H-4′b), 1.55 (2H, m, H-2″), 1.55 (2H, m, H-3″), 1.30 (2H, m, H-4″), 1.24 (3H, s, H-10′), 1.16 (3H, s, H-9′), 1.08 (3H, s, H-8′), 0.88 (3H, t, *J* = 6.6 Hz, H-5″); ^13^C NMR (CDCl_3_, 100 MHz) δ 153.4 (C-5), 152.6 (C-1), 142.9 (C-3), 109.5 (C-4), 108.4 (C-2), 105.1 (C-6), 75.8 (C-3′), 51.4 (C-2′), 46.4 (C-7′), 42.4 (C-4′), 39.2 (C-6′), 35.6 (C-1″), 34.3 (C-5′), 32.2 (C-9′), 31.5 (C-4″), 30.8 (C-2″-3″), 20.1 (C-1′), 19.7 (C-10′), 19.6 (C-8′), 14.0 (C-5″); ESIMS *m*/*z* 443 [M+H]^+^; HRESIMS *m*/*z* [M+H]^+^ 443.1439 (calcd for C_21_H_32_IO_2_, 443.1447).

Compound **5**. Colorless amorphous solid. [α]_D_ = 0. ^1^H NMR (CD_3_OD, 400 MHz) δ 6.15 (1H, s, H-4), 6.04 (1H, s, H-2), 2.68 (1H, dd, *J* = 16.7, 4.7 Hz, H-1′a), 2.40 (2H, t, *J* = 7.7 Hz, H-1″), 2.25 (1H, dd, *J* = 16.7, 13.4 Hz, H-1′b), 1.89 (1H, d, *J* = 10.3 Hz, H-4′a), 1.61 (1H, overlapped, H-5′a), 1.55 (4H, overlapped, H-2″, H-3″), 1.51 (1H, overlapped, H-4′b), 1.49 (1H, overlapped, H-2′), 1.42 (2H, H-5′b, H-6′a, overlapped), 1.33 (1H, overlapped, H-6′b), 1.32 (2H, overlapped, H-4″), 1.17 (3H, s, H′-10), 1.08 (3H, s, H′-9), 0.94 (3H, s, H′-8), 0.89 (3H, s, H-5″); ^13^C NMR (CD_3_OD, 100 MHz) δ 153.8 (C-5), 152.7 (C-1), 139.4 (C-3), 106.2 (C-2), 105.0 (C-6), 104.2 (C-1), 71.8 (C-3′), 41.6 (C-2′), 39.5 (C-6′), 37.3 (C-4′), 33.9 (C-1″), 32.3 (C-7′), 30.0 (C-9′), 29.7 (C-4″), 29.3 (C-2″-3″), 24.1 (C-10′), 19.0 (C-8′), 15.8 (C-1′, C-5′), 11.7 (C-5″); ESIMS *m*/*z* 317 [M+H]^+^; HRESIMS *m*/*z* [M+H]^+^ 317.2469 (calcd for C_21_H_33_O_2_, 317.2481).

### 3.2. Antibacterial Activity Testing

The bacterial strains used in this study are listed in Table 1. Compounds were resuspended in DMSO at a final concentration of 8 mg/mL and subsequently diluted in the culture medium. MIC and MBC values of the compounds were determined using the microdilution broth method using the cation-adjusted Mueller Hinton broth (CAMHB, Becton Dickinson) as recommended by the Clinical Laboratory Standards Institute (CLSI). Bacterial strains were pre-cultivated for 18 h at 37 °C in 15 mL tubes containing 3 mL of CAMHB and then inoculated to a final concentration of 5 × 10^5^ CFU/mL in CAMHB in each well. Each compound was tested over a concentration range of 64–0.125 μg/mL. MICs and MBCs were determined after 24 h of incubation at 37 °C. The MIC was defined as the lowest concentration that completely inhibited bacterial growth as detected by the unaided eye.

### 3.3. Toxicity Assay

The hemolytic activity has been tested on human erythrocytes from healthy 0 Rh-negative, A+, and B+ donors, as previously described [58]. Briefly, a suspension of 5% erythrocytes in PBS was incubated for 30 min at 37 °C in the presence of increasing concentrations (from 1 µg/mL to 64 µg/mL) of test compound dissolved in DMSO. The highest concentration of DMSO (0.4% *v*/*v*) was also tested to rule out any hemolytic activity of the solvent. After incubation with the different compounds, the erythrocytes were removed by centrifugation at 500× *g* for 8 min, and hemoglobin release was determined by measuring the absorbance at 540 nm (A_540_) of the supernatant. Assays were conducted in triplicate. Hemolysis was expressed in percentage, relative to 0% of lysis of erythrocytes in the negative control (blank with PBS) and 100% of lysis of erythrocytes in the presence of 0.2% Triton X-100 (total lysis), according to the following formula:

$$\mathrm{Hemolysis}\;(\%)=\frac{A_{sample}-A_{blank}}{A_{total\;lysis}-A_{blank}}\times 100$$

where *A_sample_*, *A_blank_*, and *A_total lysis_* correspond to A_540_ of the sample treated with phytocannabinoid compounds, the negative control (erythrocytes in PBS, 0% lysis), and the positive control (erythrocytes lysed in PBS with 0.2% Triton X-100, 100% lysis), respectively. The hemolytic activity was tested on samples from three donors with A+, B+, and 0- blood groups. Average and SD were calculated on the hemolytic activity determined for the three blood groups.

To test the toxicity in *G. mellonella* larvae, 3.2 mg/kg of the tested compounds were injected into *G. mellonella* larvae, as previously reported [27]. Larvae were incubated at 37 °C, and their survival was monitored every 24 h for 3 days. Larvae melanized and/or unresponsive to multiple tactile stimulations were considered dead. Each experimental condition for the toxicity test included groups of 30 larvae.

### 3.4. In silico Studies

#### 3.4.1. Ligand and Panel Preparation

The molecular structures of compounds **1**–**5** were built using the 2D Sketcher tool available in Maestro (Schrödinger Suite 2024-1) [59]. The compounds were then prepared with LigPrep [60] to assign the correct bond order and protonation state.

The advanced search panel of the Protein Data Bank (https://www.rcsb.org/, accessed on 2 December 2024) was used to retrieve the PDB codes of the proteins belonging to *S. aureus*, *B. spizizenii*, and *E. faecium*. Exploiting an automated workflow developed by our team [30], the experimental structures were downloaded, prepared, and parametrized, and the grid boxes required for the next molecular docking experiments were generated. Specifically, non-essential elements of each protein crystal structure, such as ions, solvents, and crystallization buffer components, were removed. Afterward, using the Protein Preparation Wizard tool [61,62], hydrogens were added, bond orders were corrected, and protonation states at physiological pH were determined. If the original crystal structure contained a ligand, its coordinates were used to identify the binding cavity with SiteMap [63]; otherwise, the software scanned the protein surface to determine the five most probable binding sites, selecting the highest-scoring one for subsequent steps. Once the binding site was defined, the corresponding coordinates were used to generate the molecular docking grid, with a 10–20 Å buffer in each direction, depending on the case, and a grid spacing of 1.0 Å.

#### 3.4.2. Inverse Virtual Screening

The molecular docking calculations were performed with Glide [64,65,66,67] in XP mode with enhanced sampling; an initial set of 10,000 poses was generated for each ligand, with the top 800 submitted to preliminary energy minimization. A van der Waals radii scaling factor of 0.8 and a partial charge cutoff of 0.15 were used. Following the minimization step, the 50 highest-ranked poses for each ligand were retained. The obtained predicted binding energies were normalized using 15 “decoy” molecules that shared similar physical-chemical properties (e.g., MW, hydrogen bond donor/acceptor) with compounds **1**–**5** but had different chemical structures. The same IVS procedure was followed, and the average energy of these decoys was used to normalize the predicted binding affinity of each compound for every target in the panel, generating a parameter called “V”. In detail, this parameter is calculated as follows:

$$V=\frac{V_{0}}{V_{R}}$$

where, for a given target, V_0_ is the binding affinity for the test compound, and V_R_ is the average binding affinity value of the decoys. After the normalization step, the putative interacting proteins were selected by applying some filters (i.e., a V value above 1.0 and a ligand binding affinity below −6.0 kcal/mol). Once the list of proteins is narrowed down, a visual inspection is carried out as the last evaluation step to determine whether the considered ligand interacts with key amino acids inside the protein binding cavity.

## 4. Conclusions

The increasing prevalence of MDR bacterial infections underscores the urgent need for innovative antimicrobial strategies. In this study, we have further supported the broad-spectrum antibacterial potential of natural and (semi)synthetic phytocannabinoids, including some species that had not been previously evaluated for their susceptibility to this class of metabolites. Antibacterial activity was documented for Gram-positive species.

Exploiting Inverse Virtual Screening (IVS) for the first time, we successfully singled out their putative molecular targets, reporting interactions with key bacterial enzymes involved in metabolic and defense pathways. These findings suggest that these phytocannabinoids likely exert their antibacterial effects via multi-target inhibition, interfering with multiple essential bacterial pathways.

Our study also represents a significant breakthrough in IVS, broadening its scope beyond human protein targets. The insights gained through this method lay the groundwork for more complex and integrative studies. Indeed, further experimental validation of the identified targets is essential to confirm these predictions and refine the molecular mechanisms underlying phytocannabinoid antibacterial activities. Moreover, strain-specific proteins are required to better discriminate different activities and design selective and more potent compounds.

Overall, our findings support the continued investigation of phytocannabinoids as potential antibacterial agents, emphasizing the need for integrative approaches combining computational modeling, biochemical assays, and microbiological studies.

## Figures and Tables

**Table 1:** Bacterial strains.

	Strain	Gram	Country	Year	Isolation Source	Resistance	References
*Bacillus subtilis* subsp. *spizizenii*	ATCC 6633	+	ns	1976	desert soil	NIS	American Type Culture Collection
*Escherichia coli*	MG1655	-	ns	1895	laboratory strain	ns	American Type Culture Collection
*Enterococcus faecalis*	ATCC 29212	+	ns	ns	urine	ns	American Type Culture Collection
*Enterococcus faecalis*	ATCC 700802	+	USA	1987	blood	VAN (*vanB*), GEN	[24]
*Enterococcus faecium*	ATCC 19434 ^T^	+	ns	ns	ns	ns	[25]
*Enterococcus faecium*	BM4147	+	ns	ns	ns	VAN (*vanA*)	[26]
*Staphylococcus aureus*	ATCC 25923	+	USA	1945	ns	MSSA	American Type Culture Collection
*Staphylococcus aureus*	ATCC 43300	+	USA	ns	ns	MRSA	American Type Culture Collection

Abbreviations: GEN, gentamicin; MRSA, methicillin-resistant *S. aureus*; MSSA, methicillin-sensitive *S. aureus*; NIS, nisin; VAN, vancomycin; ns, not specified; ^T^ strain type.

**Table 2:** Antimicrobial activity of compounds **1**–**5.**

				MIC (µg/mL)				MBC (µg/mL)
Cpd	*S. aureus* ATCC 25923	*S. aureus* ATCC 43300	*E. faecalis* ATCC 29212	*E. faecalis* ATCC 700802	*E. faecium* ATCC 19434	*E. faecium* BM4147	*B. spizizenii* DSM 347	*S. aureus* ATCC 25923
**1**	2	2	4	4	1	2	2	8
**2**	2	8	8	8	2	1	4	8
**3**	2	1	2	2	1	1	1	4
**4**	8	>64	4	8	2	1	4	>64
**5**	2	4	4	4	2	1	2	8

**Table 3:** The UniProt IDs of the best-interacting targets for compounds **1**–**5**.

Compound	Target Proteins
**1**	I3U4H4, P50870, Q3XX76, Q3Y316, Q9WVY4
**2**	I3U4H4, P50870, Q3XX76, Q3Y316
**3**	A0A1S8KJG1, I3U4H4, P50870, Q3XX76
**4**	P50870
**5**	A0A1S8KJG1, I3U4H4, P50870

## Data Availability

The original contributions presented in this study are included in the article/Supplementary Materials. Further inquiries can be directed to the corresponding author(s).

## Supplementary Materials

The following supporting information can be downloaded at: https://www.mdpi.com/article/10.3390/plants14131901/s1, Figure S1. Toxicity of selected phytocannabinoids in *G. mellonella*; Figure S2–S11. NMR spectra of compounds **1**–**5**; Table S1. Hemolytic activity (%) of selected phytocannabinoids, SMILES of the tested compounds, SMILES of the decoy compounds, and *S. aureus* additional data; Table S2. Targets in common between compounds **1**, **2**, and **3**; Table S3. Binding affinity and V value of compound **4** towards the targets listed in Table S2; Table S4. Results of compound **5** towards the targets listed in Table S2; Molecular docking results on *E. faecium*; Table S5. The putative targets highlighted for compound **1** sorted according to their UniProt ID; Table S6. The putative targets highlighted for compound **2** sorted according to their UniProt ID; Table S7. The putative targets highlighted for compound **3** sorted according to their UniProt ID; Table S8. The putative targets highlighted for compound **4** sorted according to their UniProt ID; Table S9. The putative targets highlighted for compound **5** sorted according to their UniProt ID; Table S10. The UniProt IDs of the most promising protein partners for each compound predicted by IVS.
