# Supplementary material for: Exploring the Potential of Phytocannabinoids Against Multidrug-Resistant Bacteria
Source: Plants (Basel). 2025 Jun 20;14(13):1901. doi: 10.3390/plants14131901 (PMC12251955; doi:10.3390/plants14131901)
Supplement: Supplementary file 1 [file plants-14-01901-s001.zip › plants-3634720-supplementary.pdf]

SUPPLEMENTARY MATERIAL

Exploring the Potential of Phytocannabinoids Against Multidrug-Resistant Bacteria

Carmina Sirignano [1], Simona De Vita [2], Ernesto Gargiulo [1], Massimiliano Lucidi [3], Daniela Visaggio [3], Maria Giovanna Chini [4], Gianluigi Lauro [2], Giuseppina Chianese [1,*], Paolo Visca [3], Giuseppe Bifulco [2,*] and Orazio Taglialatela-Scafati [1]

[1] Department of Pharmacy, University of Naples Federico II, Via Domenico Montesano 49, 80131 Napoli, Italy; carmina.sirignano@unina.it (C.S.); ernesto.gargiulo@unina.it (E.G.); scatagli@unina.it (O.T.-S.)
[2] Department of Pharmacy, University of Salerno, Via Giovanni Paolo II 132, 84084 Fisciano, Italy; sdevita@unisa.it (S.D.V.); glauro@unisa.it (G.L.)
[3] Department of Science, Roma Tre University, Viale Guglielmo Marconi 446, 00146 Rome, Italy; massimiliano.lucidi@uniroma3.it (M.L.); daniela.visaggio@uniroma3.it (D.V.); paolo.visca@uniroma3.it (P.V.)
[4] Department of Biosciences and Territory, University of Molise, Contrada Fonte Lappone, 86090 Pesche, Italy; mariagiovanna.chini@unimol.it
* Correspondence: g.chianese@unina.it (G.C.); bifulco@unisa.it (G.B.); Tel.: +39-081674125 (G.C.); +39-089969741 (G.B.)

Table of contents

Figure S1. Toxicity of selected phytocannabinoids in *G. mellonella*. 3

Figure S2. ^{1}H NMR spectrum (500 MHz) of compound 1 and 2 in $CDCl_3$. 3

Figure S3. ^{1}H NMR spectrum (500 MHz) of compound 3 in $CDCl_3$. 4

Figure S4. ^{1}H NMR spectrum (500 MHz) of compound 4 in $CDCl_3$ 4

Figure S5. 2D NMR COSY spectrum (500 MHz) of compound 4 in $CDCl_3$ 5

Figure S6. 2D NMR HMBC spectrum (500 MHz) of compound 4 in $CDCl_3$ 5

Figure S7. 2D NMR HSQC spectrum (500 MHz) of compound 4 in $CDCl_3$ 6

Figure S8. ^{1}H NMR spectrum (500 MHz) of compound 5 in CD_3OD. 6

Figure S9. 2D NMR COSY spectrum (500 MHz) of compound 5 in CD_3OD. 7

Figure S10. 2D NMR HMBC spectrum (500 MHz) of compound 5 in CD_3OD. 7

Figure S11. 2D NMR HSQC spectrum (500 MHz) of compound 5 in CD_3OD. 8

Table S1. Hemolytic activity (%) of selected phytocannabinoids 8

SMILES of the tested compounds 9

SMILES of the decoy compounds 9

***S. aureus* additional data** 10

Table S2. Targets in common between compounds 1, 2, and 3. 10

Table S3. Binding affinity and V value of compound 4 towards the targets listed in Table S2................ 11

Table S4. Results of compound 5 towards the targets listed in Table S2... 11

Molecular docking results on *E.faecium* 12

Table S5. The putative targets highlighted for compound 1 sorted according to their UniProt ID.......... 12

Table S6. The putative targets highlighted for compound 2 sorted according to their UniProt ID.......... 12

Table S7. The putative targets highlighted for compound 3 sorted according to their UniProt ID.......... 13

Table S8. The putative targets highlighted for compound 4 sorted according to their UniProt ID.......... 13

Table S9. The putative targets highlighted for compound 5 sorted according to their UniProt ID.......... 13

Table S10. The UniProt ID of the most promising protein partners for each compound predicted by IVS. 14

Figure S1. Toxicity of selected phytocannabinoids in *G. mellonella*. Fifth-instar larvae of *G. mellonella* (n=30 for each experimental group) were injected with saline or 3.2 mg/kg of the indicated compounds. After injection, larvae were incubated at 37°C, and their survival was monitored every 24 h for 3 days. Asterisks (*$P<0.05$, **$P<0.01$, ****$P<0.0001$) indicate statistically significant differences determined with the log-rank test between the survival plots of larvae injected with saline and the indicated phytocannabinoid

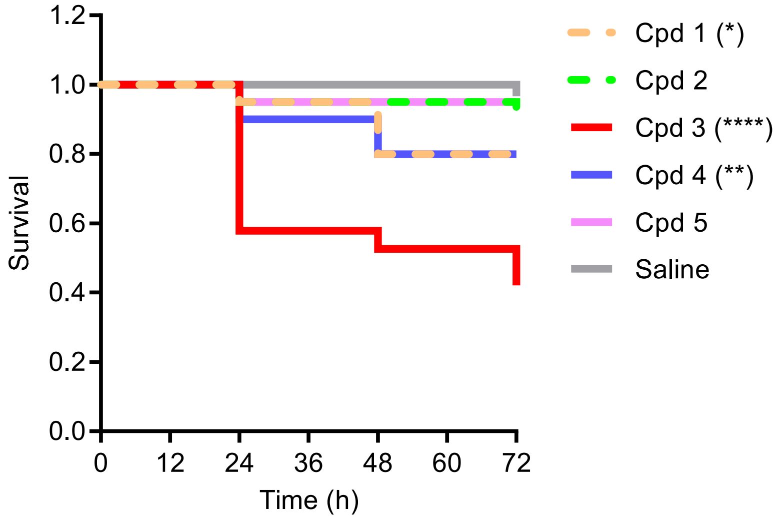

Figure S2. ^{1}H NMR spectrum (500 MHz) of compound **1** and **2** in $CDCl_3$.

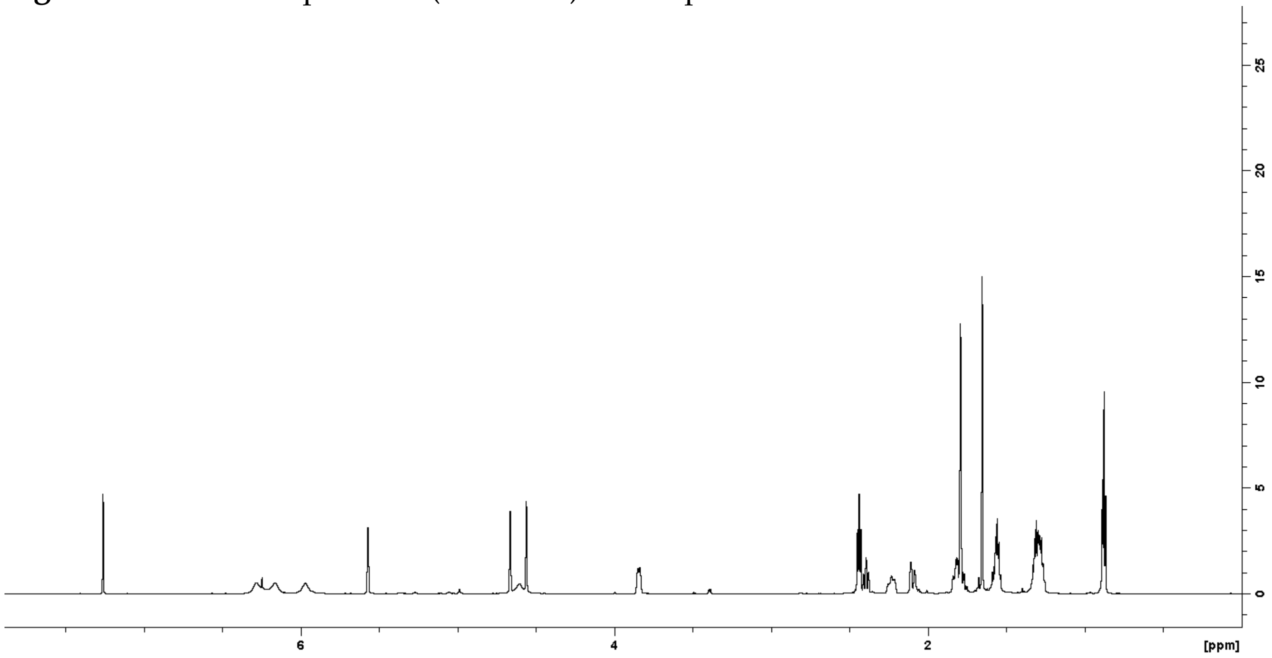

Figure S3. ^{1}H NMR spectrum (500 MHz) of compound **3** in $CDCl_3$.

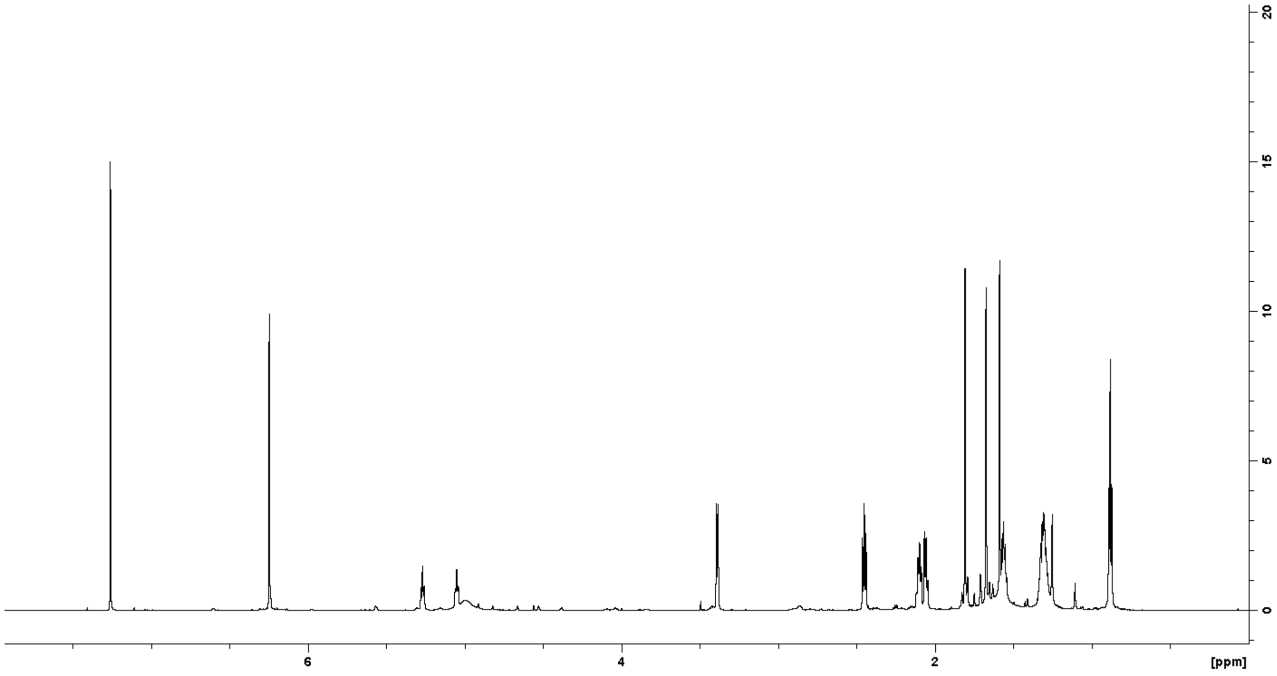

Figure S4. ^{1}H NMR spectrum (500 MHz) of compound **4** in $CDCl_3$.

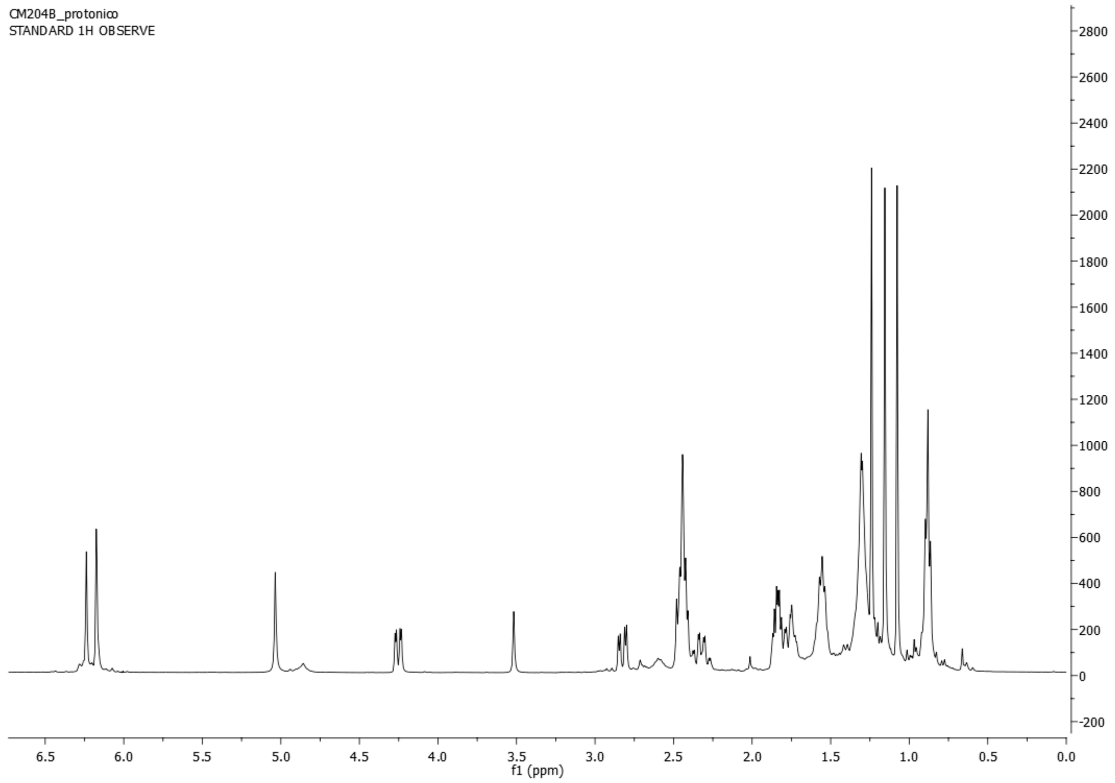

Figure S5. 2D NMR COSY spectrum (500 MHz) of compound **4** in $CDCl_3$.

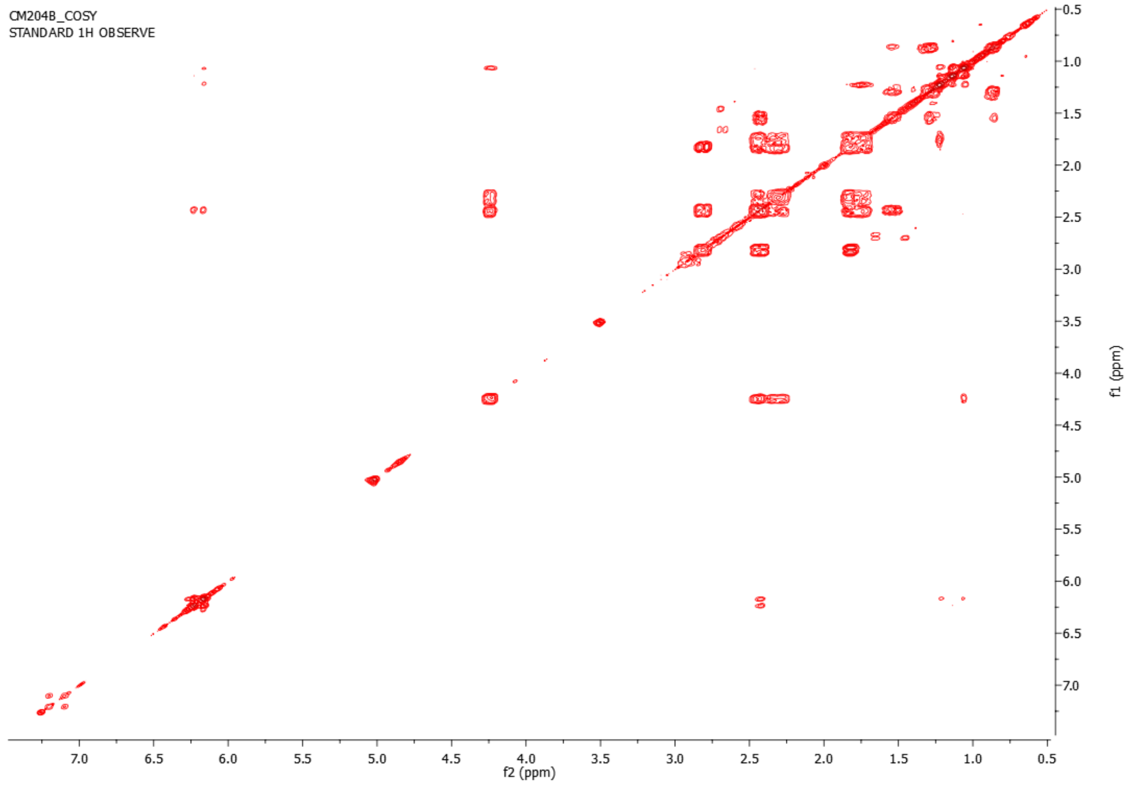

Figure S6. 2D NMR HMBC spectrum (500 MHz) of compound **4** in $CDCl_3$.

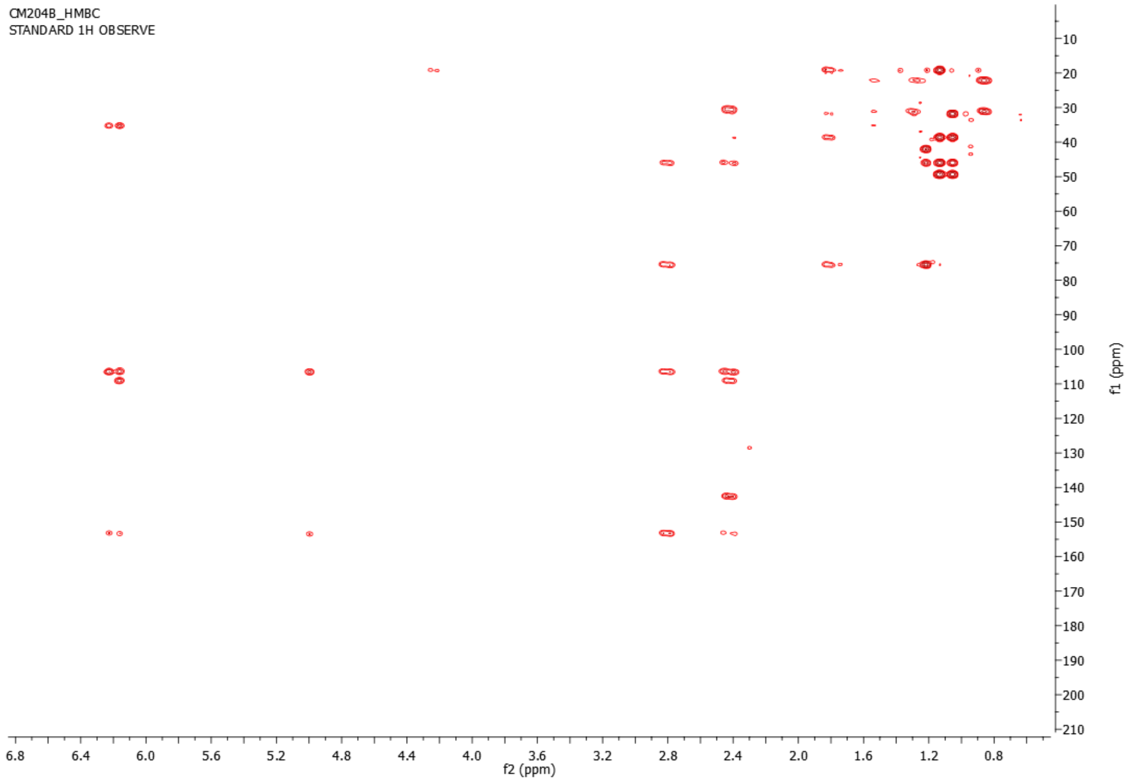

Figure S7. 2D NMR HSQC spectrum (500 MHz) of compound **4** in $CDCl_3$.

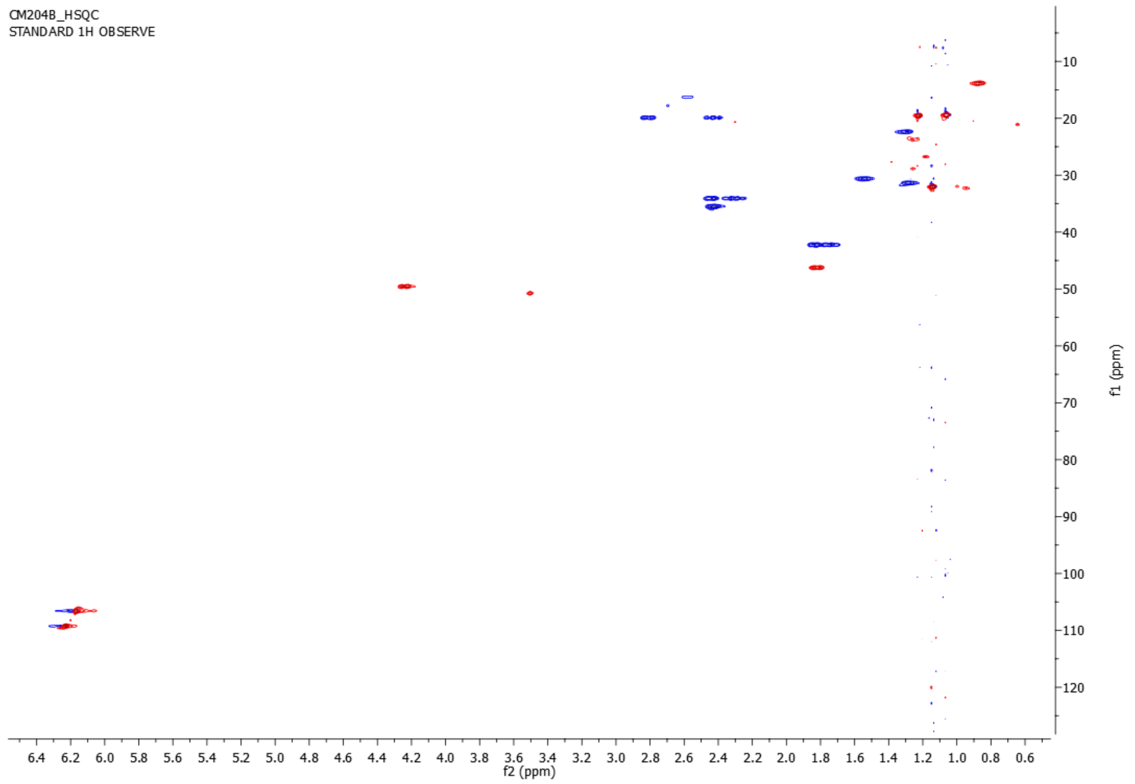

Figure S8. ^1H NMR spectrum (500 MHz) of compound **5** in CD_3OD.

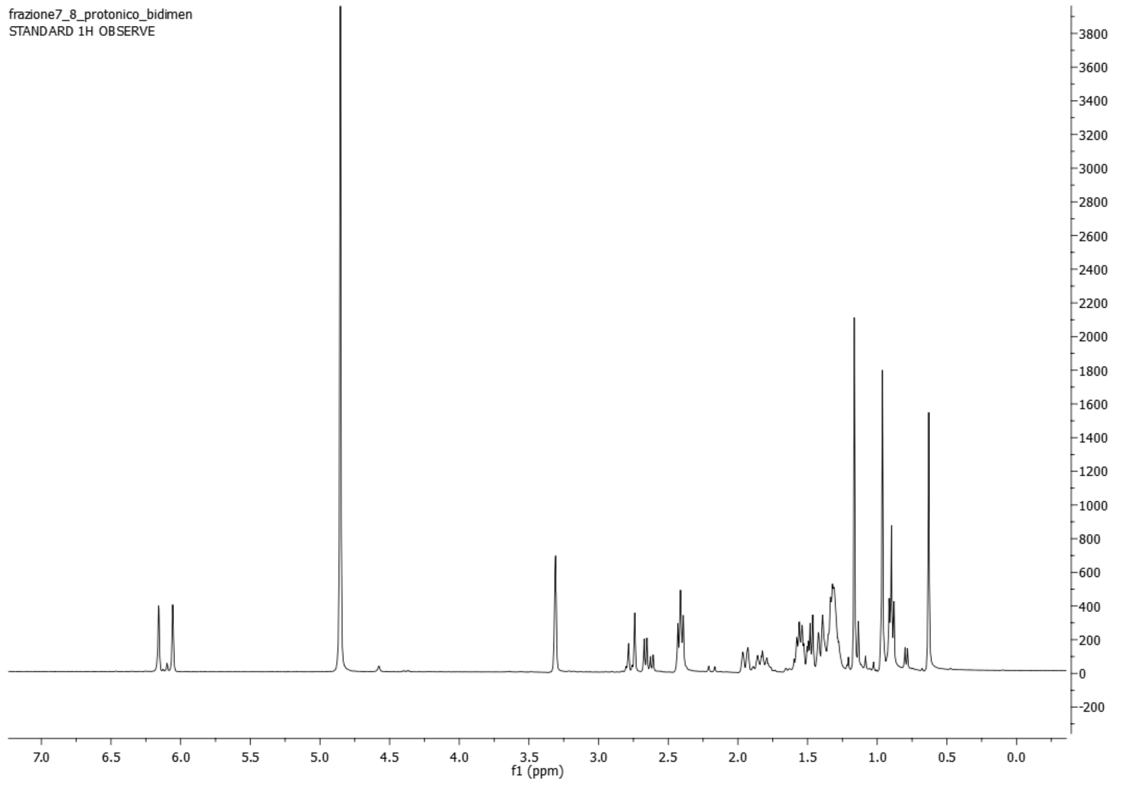

Figure S9. 2D NMR COSY spectrum (500 MHz) of compound **5** in CD_3OD.

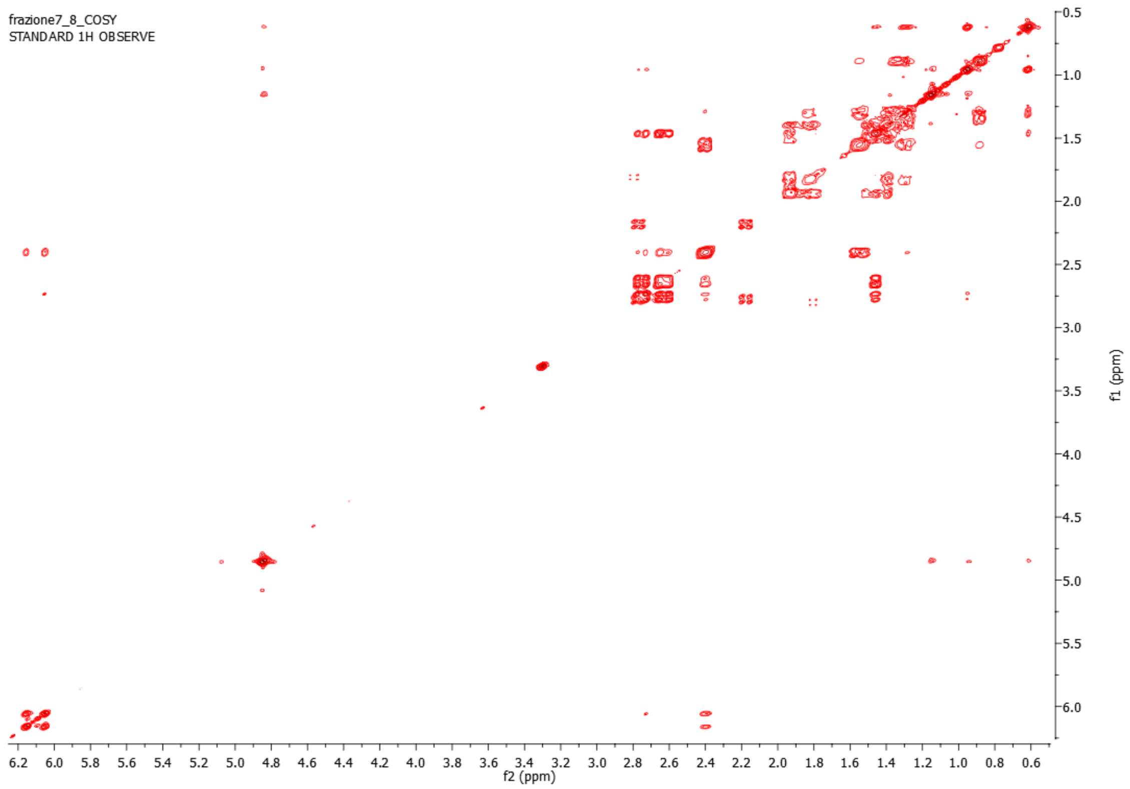

Figure S10. 2D NMR HMBC spectrum (500 MHz) of compound **5** in CD_3OD.

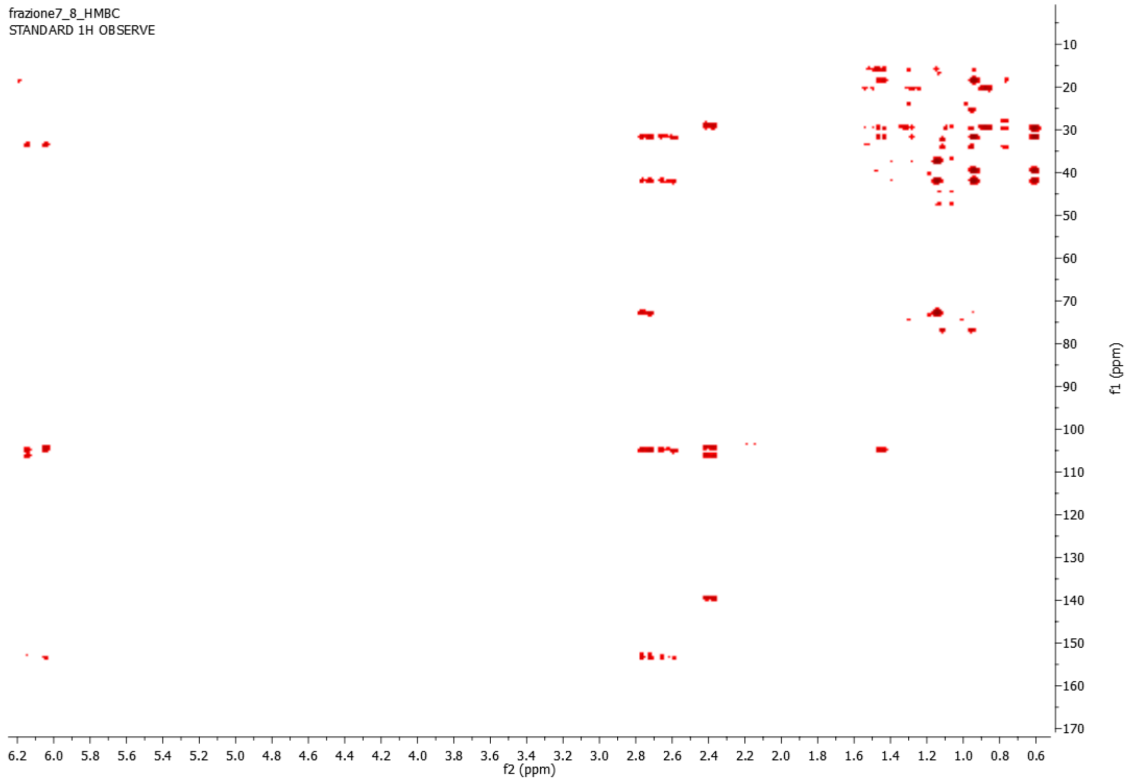

Figure S11. 2D NMR HSQC spectrum (500 MHz) of compound **5** in CD_3OD.

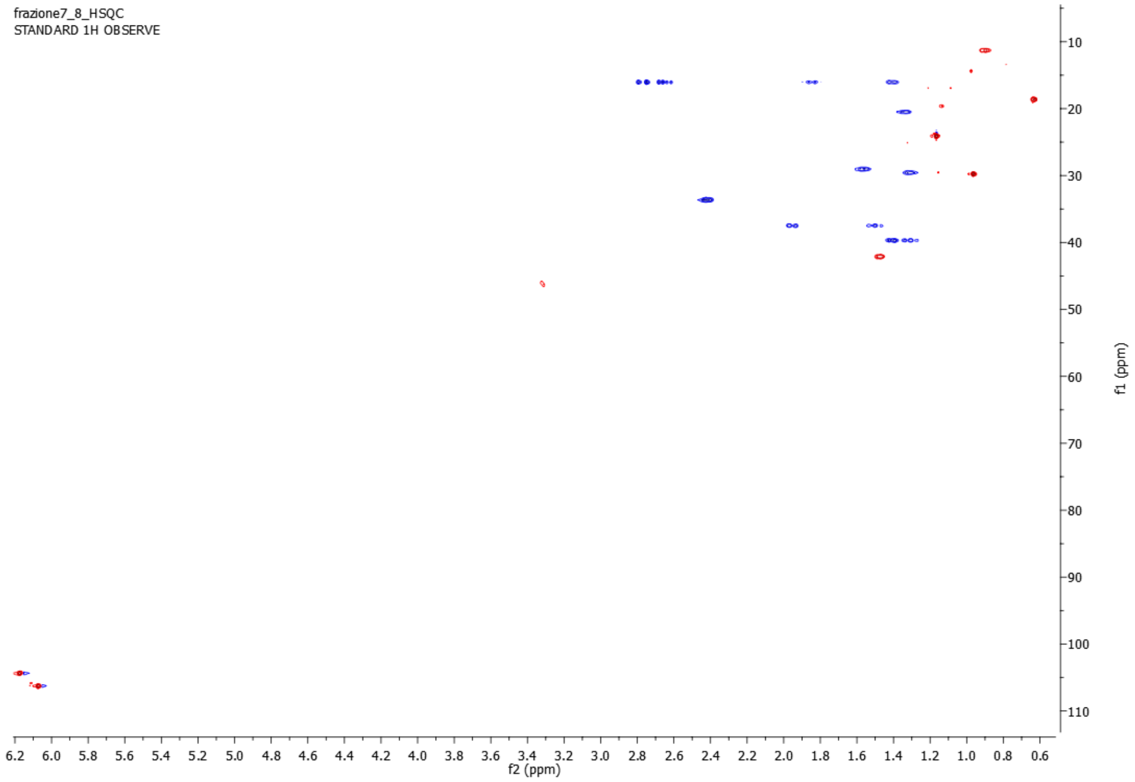

Table S1. Hemolytic activity (%) of selected phytocannabinoids

Cpd	Blood group of erythrocytes			Average ± SD
	A+	B+	0-	
**1**	0.48	1.06	0.28	0.61±0.40
**2**	0.52	0.35	0.29	0.39±0.12
**3**	1.41	1.18	2.34	1.64±0.62
**4**	0.48	0.31	0.20	0.33±0.14
**5**	0.81	0.74	0.42	0.66±0.21

SMILES of the tested compounds

1 CC1=C[C@H]([C@H](C(C)=C)CC1)c2c(O)cc(cc2O)CCCCC

2 CC1=C[C@@H]([C@@H](C(C)=C)CC1)c2c(O)cc(cc2O)CCCCC

3 CCCCCc1cc(O)c(c(c1)O)C/C=C(\C)CCC=C(C)C

4 CCCCCc(cc1O)cc(O2)c1C[C@@H]([C@]23C)C(C)(C)[C@H](I)CC3

5 CCCCCc(cc1O)cc(O2)c1C[C@@H]([C@@]23C)C(C)(C)CCC3

SMILES of the decoy compounds

c1cccc([n+]2C)c1cc(c2c34)[nH]c3cc(Cl)c(Br)c4 CHEMBL90690

c1cccc([n+]2C)c1c(Cl)c(c23)[nH]c4c3cc(cc4)-c5ccccc5 CHEMBL92462

c1cc(Cl)ccc1-c2[nH]c(c(c23)CCCC3)-c4ccc(Cl)cc4 CHEMBL140726

c1c(Cl)ccc([n+]2C)c1cc3[nH]c(c4c23)ccc(Br)c4 CHEMBL191791

C1CCCCC1N(C(C)C)C(=[N+](C)C)NCc2ccc(Cl)cc2 CHEMBL251070

c1cccc([n+]2C)c1c(Cl)c(c23)[nH]c4c3ccc(Br)c4 CHEMBL420296

c1cc(Cl)cc([n+]2C)c1cc3[nH]c(c4c23)ccc(Br)c4 CHEMBL450632

c1cccc([n+]2C)c1c(Cl)c(c23)[nH]c4c3ccc(Br)c4 CHEMBL467746

c1c(Br)ccc([n+]2C)c1c(Cl)c(c23)[nH]c4c3cccc4 CHEMBL499478

c1ccc(c2c13)[nH]cc2[C@@H]4[C@@H](C3(C)C)C[C@@H](Cl)[C@@](C)([C@@H]4[N+]#[C-])C=C CHEMBL540434

c1c(Br)ccc(c12)[nH]c3c2cc[n+](c3)Cc4ccccc4 CHEMBL609912

c1cccc([n+]2C)c1cc(c2c34)[nH]c3cc(Cl)c(Br)c4 CHEMBL1179635

c1cccc([n+]2C)c1c(Cl)c(c23)[nH]c4c3cc(cc4)-c5ccccc5 CHEMBL1179660

c1c(Br)ccc(c12)[nH]c3c2cc[n+](c3)Cc4ccccc4 CHEMBL1198661

c1c(Br)ccc(c12)[nH]c3c2c(C)c4c(c3C)cc[n+](c4)C CHEMBL1818244

[C-]#[N+]C1[C@@](C)(C=C)[C@H](Cl)C[C@H](C2(C)C)C=1c3c[nH]c(c3c24)ccc4 CHEMBL2071357

c1cccc(c12)[nH]c3c2cc[n+](c3C)Cc4ccc(C(F)(F)F)cc4 CHEMBL3577746

S. aureus additional data

The complete analysis of the IVS results is reported below.

Table S2. Targets in common between compounds 1, 2, and 3. The targets are sorted according to their UniProt ID.

UniProt ID	*Protein name*	1		2		3	
		BA	V^a	BA	V^a	BA	V^a
A0A0H2X0S3	Betaine-aldehyde dehydrogenase	-6.96	1.03	-7.27	1.16	-7.9	1.04
A0A0H3JLH9	Enoyl-[acyl-carrier-protein] reductase [NADPH]	-7.74	1.32	-9.42	1.55	-9.92	1.96
A0A0H3K1U2	HMG-CoA	-8.71	1.32	-8.95	1.37	-9.25	1.47
A0A0J9X1X7	Enoyl-[acyl-carrier-protein] reductase [NADPH]	-6.75	1.11	-7.62	1.25	-8.38	1.45
A0A0J9X1Y0	Enoyl-[acyl-carrier-protein] reductase [NADPH]	-7.51	1.16	-7.76	1.24	-9.75	1.55
A0A380DQV1	Cytochrome P450 protein	-6.50	1.16	-7.27	1.32	-7.51	1.34
A9JQL9	4,4'-diapophytoene synthase	-9.45	1.21	-8.38	1.15	-8.05	1.22
P0A017	Dihydrofolate reductase	-7.26	1.13	-7.53	1.10	-8.64	1.18
P0A0N4	HTH-type transcriptional regulator QacR	-6.52	1.11	-7.81	1.01	-6.9	1.10
P56740	Dihydroneopterin aldolase	-6.46	1.23	-6.25	1.19	-6.51	1.18
Q2FIA5	Coenzyme A disulfide reductase	-6.73	1.04	-6.96	1.13	-7.31	1.15
Q2G0I4	FAD-containing oxidoreductase	-6.54	1.11	-7.05	1.16	-7.17	1.19
Q2G1L5	Bifunctional metallophosphatase/5'-nucleotidase	-6.90	1.09	-6.16	1.13	-7.63	1.20
Q2YUF3	Ketol-acid reductoisomerase	-7.93	1.44	-7.22	1.32	-7.63	1.38
Q6GDK5	Pantothenate synthetase	-6.49	1.09	-6.69	1.21	-7.78	1.40
Q6GI75	Enoyl-[acyl-carrier-protein] reductase [NADPH] FabI	-7.29	1.12	-7.61	1.17	-7.73	1.38
Q7A0G9	Adenylosuccinate lyase	-6.65	1.06	-6.53	1.04	-6.69	1.07
Q9FD87	HMG-CoA synthase	-7.84	1.29	-8.54	1.41	-6.95	1.14

BA: predicted Binding affinity; **V^a:** Calculated V value. Please note that the average binding affinity of the decoys used to calculate the V value is strictly dependent on the PDB structure considered, and multiple PDB structures could be available for a single UniProt ID.

Table S3. Binding affinity and V value of compound 4 towards the targets listed in Table S2. The targets are sorted according to their UniProt ID.

UniProt ID	*Protein name*	Binding Affinity	V[a]
A0A0H3JLH9	Enoyl-[acyl-carrier-protein] reductase [NADPH]	-7.58	1.27
A0A0J9X1Y0	Enoyl-[acyl-carrier-protein] reductase [NADPH]	-7.51	1.24
A0A0J9X1X7	Enoyl-[acyl-carrier-protein] reductase [NADPH]	-7.63	1.20
A9JQL9	4,4'-diapophytoene synthase	-8.17	1.12
P0A017	Dihydrofolate reductase	-8.37	1.11

a: Please note that the average binding affinity of the decoys used to calculate the V value is strictly dependent on the PDB structure considered, and multiple PDB structures could be available for a single UniProt ID.

Table S4. Results of compound 5 towards the targets listed in Table S2. The targets are sorted according to their UniProt ID.

UniProt ID	*Protein name*	Binding Affinity	V[a]
A0A0H3JLH9	Enoyl-[acyl-carrier-protein] reductase [NADPH]	-7.79	1.30
A0A0H3K1U2	HMG-CoA	-7.63	1.14
A0A0J9X1X7	Enoyl-[acyl-carrier-protein] reductase [NADPH]	-7.61	1.19
A0A0J9X1Y0	Enoyl-[acyl-carrier-protein] reductase [NADPH]	-7.47	1.23
A0A380DQV1	Cytochrome P450 protein	-7.09	1.40
A9JQL9	4,4'-diapophytoene synthase	-8.01	1.29
P0A017	Dihydrofolate reductase	-7.66	1.04

a: Please note that the average binding affinity of the decoys used to calculate the V value is strictly dependent on the PDB structure considered, and multiple PDB structures could be available for a single UniProt ID.

Molecular docking results on *E.faecium*

Table S5. The putative targets highlighted for compound 1 sorted according to their UniProt ID.

	Compound 1		
UniProt ID	***Protein name***	***Binding affinity***	***V[a]***
A0A133MWI0	Probable nicotinate-nucleotide adenylyltransferase	-6.30	1.29
F1C939	Metallothiol transferase FosB	-8.08	1.17
I3U4H4	Serine hydroxymethyltransferase	-7.54	1.46
P50870	Streptogramin A acetyltransferase	-7.36	1.27
Q3XX76	ATP-dependent Clp protease proteolytic subunit	-7.48	1.27
Q3XZW8	Glutamate racemase	-6.85	1.04
Q3Y316	2-dehydropantoate 2-reductase	-6.99	1.07
Q47759	Low affinity penicillin-binding protein 5	-6.91	1.11
Q47764	Aac(6')-Ii protein	-6.01	1.01
Q9WVY4	Lincosamide nucleotidyltransferase	-6.56	1.02

a: Please note that the average binding affinity of the decoys used to calculate the V value is strictly dependent on the PDB structure considered, and multiple PDB structures could be available for a single UniProt ID.

Table S6. The putative targets highlighted for compound 2 sorted according to their UniProt ID.

	Compound 2		
UniProt ID	***Protein name***	***Binding affinity***	***V***
A0A075Q0W3	Pbp5	-6.29	1.21
E3USM3	2-dehydropantoate 2-reductase	-6.93	1.21
F1C939	Metallothiol transferase FosB	-8.12	1.18
I3U4H4	Serine hydroxymethyltransferase	-7.67	1.44
P50870	Streptogramin A acetyltransferase	-6.80	1.17
Q3XX76	ATP-dependent Clp protease proteolytic subunit	-7.70	1.31
Q3XZW8	Glutamate racemase	-6.87	1.04
Q3Y316	2-dehydropantoate 2-reductase	-6.20	1.06

Table S7. The putative targets highlighted for compound 3 sorted according to their UniProt ID.

	Compound 3		
UniProt ID	***Protein name***	***Binding affinity***	***V***
A0A075Q0W3	Pbp5	-6.85	1.32
A0A133MWI0	Probable nicotinate-nucleotide adenylyltransferase	-6.02	1.24
A0A1S8KJG1	Probable succinyl-diaminopimelate desuccinylase	-7.00	1.07
E3USM3	2-dehydropantoate 2-reductase	-7.38	1.29
F1C939	Metallothiol transferase FosB	-8.21	1.19
G5CKR9	Penicillin binding protein 5	-6.25	1.21
I3U4H4	Serine hydroxymethyltransferase	-6.44	1.25
P50870	Streptogramin A acetyltransferase	-6.99	1.22
Q3XX76	ATP-dependent Clp protease proteolytic subunit	-7.05	1.20
Q3XZW8	Glutamate racemase	-7.00	1.05
Q3Y316	2-dehydropantoate 2-reductase	-6.85	1.05
Q47764	Aac(6')-Ii protein	-6.03	1.06

Table S8. The putative targets highlighted for compound 4 sorted according to their UniProt ID.

	Compound 4		
UniProt ID	***Protein name***	***Binding affinity***	***V***
P50870	Streptogramin A acetyltransferase	-6.02	1.04

Table S9. The putative targets highlighted for compound 5 sorted according to their UniProt ID.

	Compound 5		
UniProt ID	***Protein name***	***Binding affinity***	***V***
A0A075Q0W3	Pbp5	-6.18	1.19
A0A133CK16	Serine hydroxymethyltransferase	-6.74	1.10
A0A1S8KJG1	Probable succinyl-diaminopimelate desuccinylase	-7.43	1.14
E3USM3	2-dehydropantoate 2-reductase	-6.18	1.08
F1C939	Metallothiol transferase FosB	-7.24	1.05
G5CKR9	Penicillin binding protein 5	-6.20	1.06
I3U4H4	Serine hydroxymethyltransferase	-7.25	1.36
P50870	Streptogramin A acetyltransferase	-6.70	1.16
P50870	Streptogramin A acetyltransferase	-6.12	1.04
Q3XZW8	Glutamate racemase	-6.66	1.01
Q842S4	Peptide deformylase	-6.34	1.20

Table S10. The UniProt ID of the most promising protein partners for each compound predicted by IVS.

1	2	3	4	5
A0A133MWI0	A0A075Q0W3	A0A075Q0W3	P50870	A0A075Q0W3
F1C939	E3USM3	A0A133MWI0		A0A133CK16
I3U4H4	F1C939	A0A1S8KJG1		A0A1S8KJG1
P50870	I3U4H4	E3USM3		E3USM3
Q3XX76	P50870	F1C939		F1C939
Q3XZW8	Q3XX76	G5CKR9		G5CKR9
Q3Y316	Q3XZW8	I3U4H4		I3U4H4
Q47759	Q3Y316	P50870		P50870
Q47764		Q3XX76		Q3XZW8
Q9WVY4		Q3XZW8		Q842S4
		Q3Y316		
		Q47764		
